# Meta-Analysis of Internet Gaming Disorder Prevalence: Assessing the Impacts of DSM-5 and ICD-11 Diagnostic Criteria

**DOI:** 10.3390/ijerph21060700

**Published:** 2024-05-29

**Authors:** Ruoyu Zhou, Nobuaki Morita, Yasukazu Ogai, Tamaki Saito, Xinyue Zhang, Wenjie Yang, Fan Yang

**Affiliations:** 1 Doctoral Program in Human Care Science, Graduate School of Comprehensive Human Sciences, University of Tsukuba, Tsukuba 305-8577, Japan; zhouruoyu86@gmail.com; 2 Department of Social Psychiatry and Mental Health, School of Medicine and Medical Sciences, University of Tsukuba, Tsukuba 305-8577, Japan; nobuakim@nifty.com (N.M.); ogai.ys@md.tsukuba.ac.jp (Y.O.); hhd02063@gmail.com (T.S.); 3 Public Health Degree Program, Faculty of Comprehensive Human Sciences, University of Tsukuba, Tsukuba 305-8577, Japan; zhuiziwwww@gmail.com; 4 The Mental Health Center, Yunnan University, Kunming 650091, China; 5 Graduate School of Letters, Arts and Sciences, Waseda University, Tokyo 162-8644, Japan; fan.yang@ruri.waseda.jp

**Keywords:** Internet gaming disorder, gaming disorder, DSM-5, ICD-11, prevalence, meta-analysis

## Abstract

With the inclusion of Internet gaming disorder (IGD) in both the DSM-5 and ICD-11, understanding the prevalence and diagnostic discrepancies is crucial for developing appropriate interventions. This study presents a meta-analysis of the prevalence of IGD based on two diagnostic criteria. We systematically searched the PubMed and Web of Science databases. A total of 22 studies were included in the final analysis. The analysis incorporated studies employing the DSM-5 and ICD-11 criteria and focused on the impact of various factors, including study location, sample characteristics, sample size, and quality score, on the reported prevalence rates using a random-effects model. The pooled prevalence of IGD is 6.7% (95% CI: 5.7–7.7%). The subgroup analysis indicated significant differences in the prevalence rates of IGD (DSM-5 criteria) and GD (ICD-11 criteria) (Q b = 38.46, *p* < 0.01). There were also significant differences in IGD prevalence rates between different scales (Q b = 54.23, *p* < 0.001). Our findings indicate that different diagnostic criteria and different assessment scales have a significant impact on the prevalence of IGD. This underscores the importance of adopting standardized methodologies to guide public health interventions. However, given the limited research based on ICD-11 diagnostic criteria, further investigation is necessary to determine the variations in prevalence rates of IGD under different diagnostic standards.

## 1. Introduction

With the advancement of technology, Internet gaming disorder (IGD) has become increasingly serious among adolescents in Asia. For susceptible individuals, unrestricted online gaming can become time-consuming, energy-draining, and highly addictive. In 2021, the prevalence of gaming disorder among Japanese youth (ages 10 to 29) was of 7.6% for males and 2.5% for females, with an overall prevalence of 5.1% [1].

The Diagnostic and Statistical Manual of Mental Disorders, Fifth Edition (DSM-5) defines IGD as a condition characterized by excessive and compulsive engagement in video games, leading to significant impairment or distress over 12 months. Excessive gaming can result in negative outcomes such as academic decline, physical health issues, and social isolation [2,3]. It is worth noting that moderate gaming has been associated with benefits such as improved cognitive functions and social skills [4].

In the context of defining IGD, the World Health Organization (WHO) employs an alternative term, “Gaming Disorder” (GD), in the 11th Revision of the International Classification of Diseases (ICD-11). According to the ICD-11, GD encompasses both online and offline gaming behavior [5].

However, the diagnostic criteria for IGD (DSM-5) and GD (ICD-11) are not entirely identical. IGD is an emerging disorder requiring further study, whereas GD signifies a formal recognition of the disorder. To diagnose IGD, the DSM-5 outlines nine criteria, including preoccupation with gaming, withdrawal symptoms, tolerance, loss of control, loss of interest in previous hobbies and entertainment, continued excessive use despite psychosocial issues, deceiving family members or therapists regarding the amount of gaming, use of gaming to escape or relieve negative moods, and significant impairment or distress. Conversely, ICD-11defines GD as a pattern of gaming behavior characterized by impaired control over gaming, increasing priority given to gaming over other activities to the extent that gaming takes precedence over other interests and daily activities, and continuation or escalation of gaming despite the occurrence of negative consequences [6]. 

Some studies suggest that the measurement tools for GD and IGD exhibit factorial invariance, indicating stability across different populations and settings [6]. Conversely, other studies report inconsistent and lower concordance between the two measures [1]. Furthermore, research has shown that there is a 73% overlap in the diagnostic criteria of IGD and GD [7], yet this partial overlap does not justify the use of these terms interchangeably, as each set of criteria captures unique aspects of gaming disorders [8].

Compared to the DSM-5 criteria, the ICD-11 reclassifies behavioral addictions such as GD as addictive disorders, thus shifting the nomenclature from “dependence” to “use disorder”. This reclassification does not imply a denial of gaming itself but is framed as a measure for early intervention in problematic gaming practices. It suggests that, with proper guidelines for engaging with Internet games as leisure and entertainment activities, these can be integrated into a lifestyle that promotes health rather than addiction [9]. The question of whether this reclassification affects the prevalence rates of IGD is a critical area for future research, examining the potential shifts in epidemiological patterns due to these changes in diagnostic criteria.

The discrepancy in the measurement tools and their criteria has implications for the perceived prevalence of pathological gaming behaviors. A lack of consensus among researchers regarding diagnostic standards may result in false positive diagnoses, inflating prevalence rates and potentially stigmatizing non-pathological gaming behaviors [10]. 

It is imperative for the clinical and research communities to strive toward a unified diagnostic framework that can accurately identify and differentiate between pathological gaming behaviors and non-pathological engagement. Such harmonization would not only refine the diagnostic process but also enhance the quality of interventions and the interpretability of outcomes across different cultural and clinical contexts [11].

### 1.1. Differences in Prevalence under Different Diagnostic Criteria

The prevalence of IGD varies significantly across studies, with recent analyses revealing rates ranging from 0.7% to 27.5% in various populations. This wide range underscores the complexity and variability in IGD, reflecting its impact across diverse demographic and geographical groups [12].

The global prevalence of gaming disorders varies significantly across studies due to methodological differences, including the choice of screening tools and sampling criteria. A systematic review and meta-analysis reported a worldwide prevalence of 3.05%, with estimates adjusted to 1.96% when considering studies that met more stringent sampling criteria. Variability in prevalence rates is also influenced by factors such as the assessment tool used, participant age and sex, and region of the study [13].

Considering the above, this study aimed to further organize issues related to gaming disorders and conduct a meta-analysis to avoid stigmatizing general users of games (players without dependency symptoms, e-sports athletes, and game industry employees). It seeks to clarify the impact of different gaming disorder diagnostic criteria (DSM-5 and ICD-11) and factors such as region on gaming disorder.

In the assessment of GD, researchers have developed various tools. The first such tool, known as the Gaming Disorder Test (GDT), is designed to evaluate the severity and consequences of gaming behavior rather than to provide a clinical diagnosis [10]. 

The second tool, the Gaming Disorder Scale for Adolescents (GADIS-A), comprises ten items that gauge the extent and impact of gaming behavior on adolescents. This scale evaluates criteria such as preoccupation with gaming, withdrawal symptoms, tolerance, and interference with personal and social functioning [14].

While the GDT is instrumental in gauging the severity and consequences of gaming habits, its recommended threshold values have not been empirically validated, which limits its use in ascertaining the prevalence of GD. Consequently, the GADIS-A is the scale currently available for effectively measuring the prevalence of GD, as it has been subject to more rigorous validation processes.

The assessment of IGD has several scales, notably the IGDT-10 and IGUESS. The IGDT-10 evaluates symptoms aligned with DSM-5 criteria [15], while IGUESS measures gaming’s cognitive and emotional effects [16]. Compared to GD, the array of IGD scales reflects its established presence in research, offering diverse tools for understanding this behavioral phenomenon.

### 1.2. The Impact of Cultural Background

The impact of cultural background on IGD is significant, as one study suggests that individualistic and collectivistic cultural orientations can influence IGD [17,18]. This study identified two profiles of Internet gamers, showing that those with fewer collectivistic tendencies exhibited higher IGD behaviors. This underscores the need for culturally tailored strategies for the prevention and intervention of IGD.

A comprehensive meta-analysis highlighted that the prevalence of GD in East Asia is notably higher than in other regions of the world, with an overall pooled prevalence of 12%. This suggests significant regional differences that could be attributed to various cultural and societal factors unique to East Asian countries [19].

Research on IGD in Japan shows that it is considered a new lifestyle-related disease among children and adolescents, indicating a high concern for game addiction owing to its psychological and social impacts. Despite Japan’s prominent gaming culture, studies on IGD are less frequent than in China and South Korea. This highlights the need for expanded research in Japan and other under-studied regions to better understand IGD’s prevalence and the impact of IGD across different cultural contexts [20].

### 1.3. The Present Study

This study conducted a meta-analysis of the prevalence of IGD based on two diagnostic criteria: DSM-5 and ICD-11. Unlike previous meta-analyses, this study excluded research that did not clearly define IGD/GD or employ related assessment tools to better distinguish between IGD and Internet addiction. The specific objectives were as follows: (1) assess the prevalence of IGD after the diagnostic criteria of ICD-11 came into effect in January 2022; (2) examine the differences in IGD prevalence across various characteristics (study location, sample size, diagnostic criteria, population type, quality score, and measurement tools). This will provide accurate data on IGD prevalence rates, aid in further identification and prevention of IGD, and inform policy and treatment research.

## 2. Method

### 2.1. Protocol and Registration

This meta-analysis followed the preferred reporting items for systematic reviews and meta-analysis (PRISMA) standards as the framework for the methodology. Before initiating the analysis, the research protocol was formally registered with the International Prospective Register of Systematic Reviews (PROSPERO:CRD42024522606) to ensure transparency and compliance with established guidelines. 

### 2.2. Search Strategy and Study Selection

To identify relevant studies, two authors independently conducted a comprehensive search across multiple databases, including PubMed and Web of Science, covering all records from January 2022 to January 2024. The following search terms with combinations of keywords were used: (“video game” OR “video gaming” OR “online game” OR “online gaming” OR “computer game” OR “computer gaming” OR “internet game” OR “internet gaming”) AND (“excessive” OR “problematic” OR “problem” OR “pathological” OR “disorder” OR “addiction” OR “addicted”) AND “prevalence”. Furthermore, the reference lists of the included studies and relevant meta-analyses or reviews were manually scrutinized for additional sources.

### 2.3. Inclusion and Exclusion Criteria

The inclusion criteria for this meta-analysis were as follows: (1) original research articles written in English; (2) studies published in peer-reviewed journals between 1 January 2022 and 1 January 2024; (3) research with a clear definition of IGD involving online gaming disorder/addiction, distinguishing it from general Internet addiction; (4) studies reporting IGD prevalence rates; and (5) research available in full text. 

The exclusion criteria were as follows: (1) studies with incomplete data or duplicate publications; (2) studies without specified DSM-5 or ICD-11 diagnostic criteria; and (3) studies focusing on specific populations (e.g., religious figures, psychiatric patients).

### 2.4. Data Extraction

Data were systematically extracted using a predefined template. Two authors independently extracted data, which were then verified by a third author, including author names, publication year, study location, population type (adolescents/adult), sample size, assessment tools, and IGD prevalence rates. For longitudinal studies, only baseline data were considered. Studies covering multiple countries/regions have listed their data separately for each location. In cases of discrepancies, an initial attempt to resolve disagreements is made through team discussion. If consensus is not achieved, a third expert arbitrator is consulted to make the final decision.

### 2.5. Quality Evaluation

To assess the quality of our meta-analysis, an 11-item checklist developed by the Agency for Healthcare Research and Quality (AHRQ) was employed [21]. The Agency for Healthcare Research and Quality was used to assess the quality of the studies:

1.  Define the source of information (survey, record review);
2.  List inclusion and exclusion criteria for exposed and unexposed subjects (cases and controls) or refer to previous publications;
3.  Indicate time period used for identifying patients;
4.  Indicate whether or not subjects were consecutive if not population-based;
5.  Indicate whether evaluators of subjective components of study were masked to other aspects of the status of the participants;
6.  Describe any assessments undertaken for quality assurance purposes (e.g., test/retest of primary outcome measurements);
7.  Explain any patient exclusions from analysis;
8.  Describe how confounding was assessed and/or controlled;
9.  If applicable, explain how missing data were handled in the analysis;
10. Summarize patient response rates and completeness of data collection;
11. Clarify what follow-up, if any, was expected and the percentage of patients for which incomplete data or follow-up was obtained.

This systematic approach ensured a thorough evaluation of study quality and bolstered the reliability of our meta-analysis findings.

### 2.6. Statistical Analyses

In the statistical analyses of our meta-analysis, we initially conducted a meta-analysis using the “meta” package in Stata 18 (StataCorp LLC, College Station, TX, USA). The heterogeneity in the studies was assessed by utilizing the Galbraith plot and classifying the I2 statistic. When significant heterogeneity was detected, further examination of the individual studies was conducted, and random effects models (Dersimonian–Laird method) were used to estimate the overall prevalence of IGD and its 95% confidence interval (CI). Prediction intervals (PIs) were estimated when there were four or more studies to predict where 95% of future estimates would lie [22,23].

Subgroup analysis was also performed on the potential influencing factors. Publication bias was examined using Egger’s test and Begg’s test, with *p* < 0.05 indicating the presence of publication bias. A sensitivity analysis was conducted to determine the studies with the greatest impact on the summary estimates and to assess the robustness of the estimates. 

## 3. Results

### 3.1. Study Characteristics

**Figure 1:**
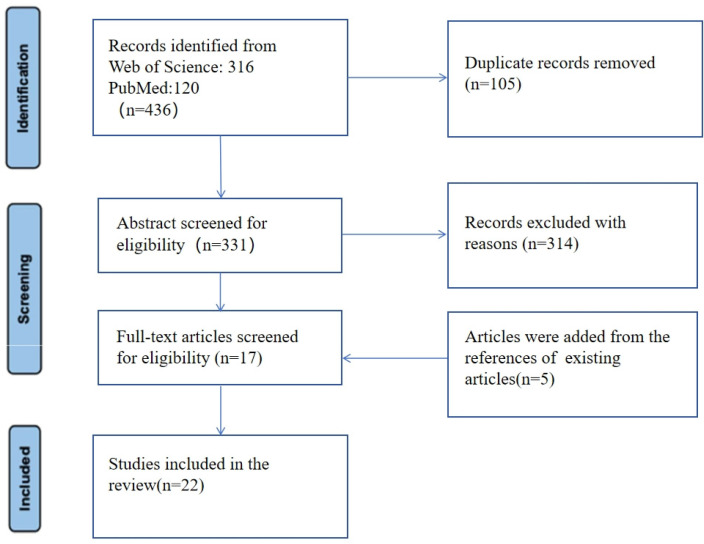
Flow diagram for study selection.

The initial literature search, conducted across two databases (PubMed: n = 120, Web of Science: n = 316), yielded a combined total of 436 records. After excluding 105 duplicates, we screened 331 records based on their titles and abstracts, as illustrated in Figure 1. During this screening process, 314 records were excluded for various reasons, including those that were irrelevant to the topic, did not meet the predetermined inclusion criteria, or presented a lack of essential data extraction. As a result, we identified 17 records for potential inclusion. To enhance review, we additionally identified five pertinent articles from the references of the remaining studies, relevant meta-analyses, and reviews published recently. This adjustment brought the total number of articles under consideration to 22 [24,25,26,27,28,29,30,31,32,33,34,35,36,37,38,39,40,41,42,43,44,45].

Table 1 summarizes the characteristics of the studies (n = 22) included in the meta-analysis. This study conducted a comprehensive analysis of multiple international research projects to explore the prevalence of IGD among adults and adolescents across various regions. The assessment tools used included IGD-20, IGDS9-SF, IGDT-10, and GADIS-A. The sample sizes ranged from 28 to 28,689 participants, with the study locations encompassing the Middle East, Asia, and Europe.

### 3.2. Prevalence of IGD

**Figure 2:**
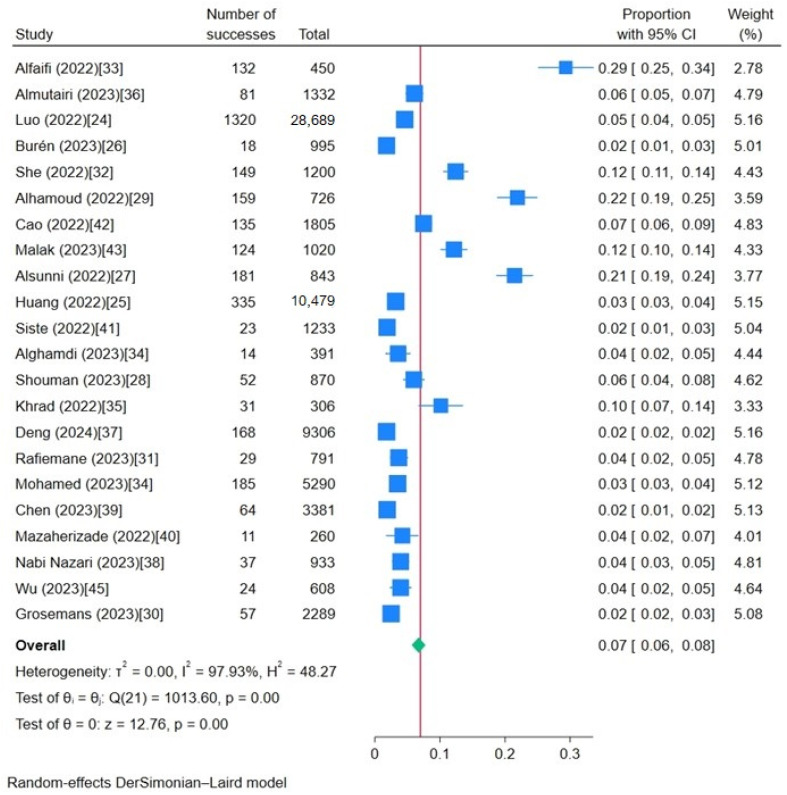
Forest plot of the prevalence of IGD (I^2^ > 75% indicates that the prevalence has high heterogeneity).

**Figure 3:**
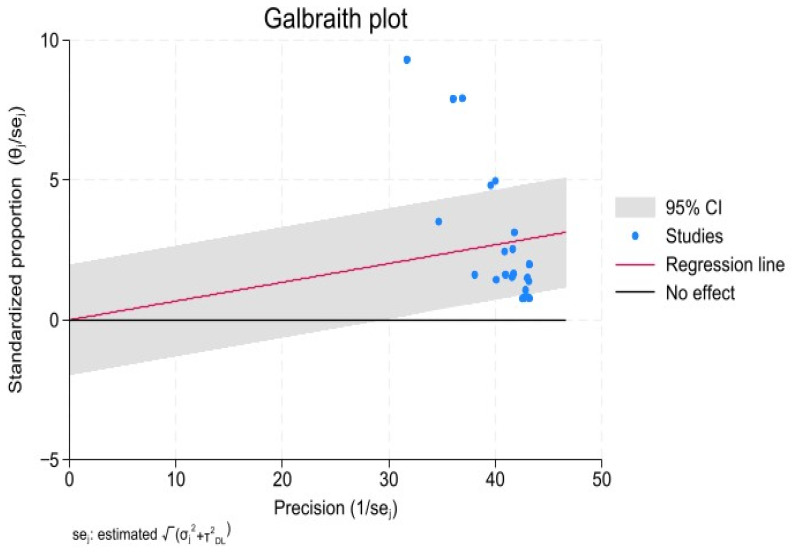
Galbraith plot of the prevalence of IGD.

The heterogeneity test revealed a pronounced disparity across the studies included in the meta-analysis, with an I^2^ value of 97.93% (*p* < 0.001) and prediction interval (0.018, 0.117) (Figure 2). Galbraith plots were used to assess the heterogeneity of the study, which showed significant heterogeneity (Figure 3). Most of the included studies in this review showed inconsistency, signifying a considerable level of heterogeneity. Accordingly, a random-effects model was used to compute the pooled prevalence of IGD. The forest plot, as shown in Figure 2, illustrates the individual study prevalence ranging from 1.8% to 29.3%, indicating a considerable range across studies. The overall aggregate prevalence of IGD was 6.7%, with a 95% confidence interval (CI) extending from 5.7% to 7.7%. 

### 3.3. Subgroup and Meta-Regression Analyses

The subgroup analysis presented in the table and corresponding figure demonstrates variability in the prevalence of IGD across different variables, although not all subgroup differences reached statistical significance (Table 2).

A total of 19 studies conducted in Asia reported a pooled prevalence of 7.5% (95% CI 6.3–8.6%), which was significantly different from the pooled prevalence of 2.6% (95% CI 1.6–3.7%) reported by three studies conducted in Europe (Q b = 37.76, *p* < 0.01). Studies with sample sizes >1000 (n = 11) and <1000 (n = 11) demonstrated significant differences in pooled prevalence rates of IGD (Q b = 6.78, *p* < 0.01). For studies with sample sizes >1000, the pooled prevalence was 4.9% (95% CI 3.8–6%). Conversely, in studies with sample sizes <1000, the pooled prevalence was higher, at 9.7% (95% CI 6.2–13.2%). A total of 10 studies investigated the prevalence of IGD among adolescents, with a pooled prevalence of 7.1% (95% CI 5.6–8.5%). A total of 12 studies assessed the adult population, reporting a pooled prevalence of 6.5% (95% CI 4.5–8.5%). The subgroup analysis showed no significant differences in the pooled prevalence rates among these three groups (*p *= 0.651).

The different scales used in these studies reflect variations in the prevalence of IGD. For example, studies using the GADIS scale reported a pooled prevalence of 3% (95% CI: 2.1–3.9%), while those employing the DSM-5 IGD checklist reported a significantly higher pooled prevalence of 13.7% (95% CI: 6.6–20.8%). The differences were statistically significant (Q b = 54.23, *p* < 0.001). According to the diagnostic assessment methods for IGD, subgroup analysis by diagnostic criteria (ICD-11, DSM-5) revealed significant differences (Q b = 38.46, *p* < 0.01). Studies based on DSM-5 criteria reported a significantly higher pooled prevalence of 7.9% (95% CI: 6.6–9.2%), while studies based on ICD-11 criteria reported a significantly lower pooled prevalence of 3% (95% CI: 2.1–3.1%). Finally, studies with QS < 9 reported a pooled prevalence of 6.1% (95% CI: 4.7–7.5%), while studies with QS > 9 showed a pooled prevalence of 7.9% (95% CI: 6.1–9.7%). There was no significant difference between the two (*p *= 0.115).

**Figure 4:**
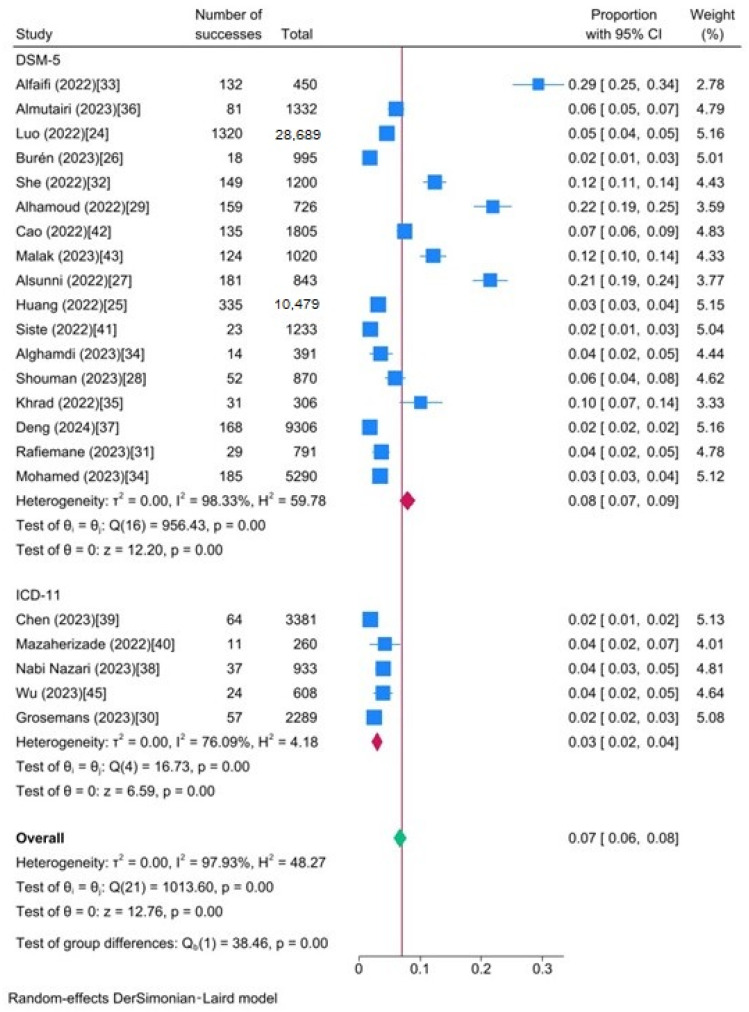
Forest plot of the prevalence of IGD between different diagnostic criteria.

Based on the results of the meta-analysis subgroup analysis provided (Figure 4), the heterogeneity of studies based on ICD-11 criteria (I^2^ = 76.09%) is lower than that of studies based on DSM-5 criteria (I^2^ = 98.33%).

### 3.4. Publication Bias

**Figure 5:**
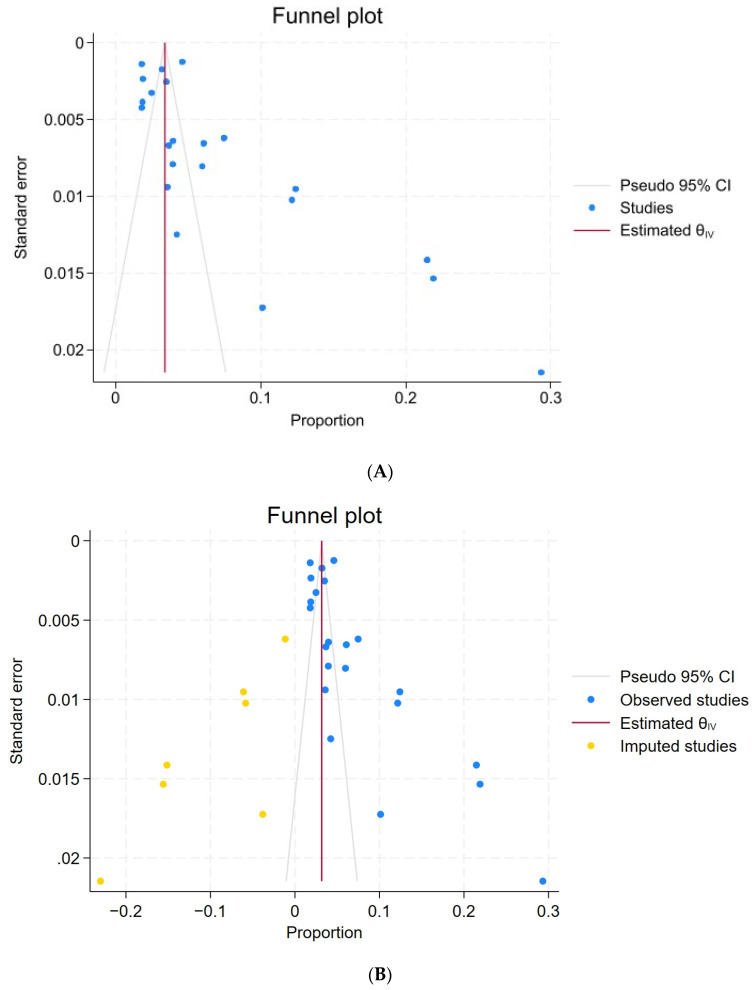
(**A**) Funnel plots to assess publication bias (original funnel plot); (**B**) Filled funnel plot to assess publication bias (studies needed to be filled in white).

The asymmetry of the funnel plot in the primary analysis (Figure 5A) indicated the presence of publication bias concerning reports on the prevalence of IGD. This suspicion was substantiated by the outcomes of Egger’s test (Z = 11.28, *p* < 0.001) and Begg’s test (Z = 3.10, *p* = 0.0019). In light of the detected publication bias, we employed the approach suggested by Wang et al. [46] and recalculated the prevalence of IGD using the trim and fill method. Consequently, 7 studies were imputed, which resulted in an updated pooled prevalence of IGD at 3.3% (95% CI: 2.1%–4.1%) (Figure 5B).

## 4. Discussion

In this meta-analysis, we found that the prevalence of IGD was 6.7%. This result contrasts somewhat with the findings of several other studies. For example, a meta-analysis including studies published before March 2020 reported a lower prevalence of IGD, at around 3.3% [47], while a more recent meta-analysis published in 2022 focusing on young people showed an increased prevalence of 9.9% [48]. 

These differences may reflect the influence of several key factors. Firstly, the increasing prevalence of the Internet is likely a major contributing factor to the rising prevalence of IGD. As Internet access becomes more widespread and convenient, a greater number of individuals are exposed to online games, thereby increasing the potential risk of IGD [49]. Furthermore, the proliferation of smartphones and tablets may also contribute to this trend, as they provide users with increased opportunities to access and engage in online gaming [50]. Secondly, the perception of the COVID-19 pandemic may also increase the risk of IGD, as it can lead to increased symptoms of depression and anxiety, which are known to be associated with IGD [51].

The prevalence of IGD in Asia is significantly higher than in Europe. This finding is consistent with the existing literature, possibly due to the higher penetration rates of Internet and video games in Asia [19].

The findings from our meta-analysis indicating different prevalence rates of IGD based on diagnostic criteria (DSM-5 and ICD-11) can be attributed to the varying stringency of these systems. The DSM-5 criteria, being broader, may capture a wider spectrum of gaming behaviors, thus reporting higher prevalence. In contrast, the stricter ICD-11 criteria emphasize more severe symptomatology and functional impairment, leading to lower reported prevalence rates [7]. 

However, there was a significant difference in the prevalence of having the disease between the different scales. Meanwhile, this study found that the prevalence obtained using scales based on the DSM-5 diagnostic criteria had a large range, while those using scales based on the ICD-11 diagnostic criteria were more stable and had lower heterogeneity. This is supported by the literature indicating that ICD-11 applies stricter thresholds compared to DSM-5, potentially leading to fewer diagnoses under ICD-11 criteria. This discrepancy may stem from the uneven weighting of the nine symptoms in the DSM-5 criteria, with some symptoms being potentially more important for diagnosing IGD. For example, loss of control over gaming behavior and preoccupation with gaming are likely to be core symptoms of IGD [52], while other symptoms such as salience of gaming and withdrawal symptoms may be secondary manifestations of IGD. When diagnostic scales measure similar concepts, setting a more lenient cut-off point will identify more individuals. However, this may lead to increased internal heterogeneity in the diagnosed group [53]. Therefore, giving items equal weight in a scale could introduce measurement errors in IGD assessment.

This study has several notable limitations. Firstly, the inclusion of only peer-reviewed articles published in English may have omitted significant findings presented in the gray literature or in other languages. This language and publication bias may limit the analyses’ comprehensiveness and the findings’ applicability. Furthermore, limited studies utilizing the ICD-11 diagnostic criteria for IGD necessitate further investigation to ensure a comprehensive global assessment. The considerable heterogeneity among the studies included in the present analysis remains evident, despite accounting for several common factors to identify the source of this variability.

Additionally, the relatively low number of studies available for different categories in our subgroup analyses could potentially affect the findings’ robustness. Future research should aim to include a more diverse set of studies, encompassing different languages and publication types, and should attempt to increase the number of studies per category in subgroup analyses.

Studies have concluded that culture can influence IGD [11]. To enhance the robustness of the findings, future research should adopt a more inclusive approach that encompasses all countries, utilizing both the DSM-5 and ICD-11 criteria to paint a more accurate picture of IGD prevalence. This will significantly contribute to the global understanding of IGD and its impact on public health.

## 5. Conclusions

This study provides a comprehensive assessment of the prevalence of IGD across different regions and populations, revealing a complex picture influenced by various factors. Varied methodological approaches, such as different scales and diagnostic criteria (ICD-11 and DSM-5), complicate direct comparisons across studies. This would facilitate accurate epidemiological assessments and inform targeted interventions. The observed variations in IGD prevalence underscore the need for localized research to address the nuanced cultural and regulatory landscapes that influence gaming behaviors. Overall, the findings call for a nuanced understanding of IGD, accounting for regional, cultural, and methodological factors to effectively address this growing concern.

## Figures and Tables

**Table 1:** Descriptive characteristics of the study included in the meta-analysis.

Study	Study Location	Sample Size	Adolescents /Adult	Scale	DSM-5 /ICD-11	QS	Prevalence
Alfaifi (2022) [33]	Saudi Arabia	450	adolescents	IGD-20	DSM-5	7	0.293
Almutairi (2023) [36]	Three Arab countries	1332	adult	IGD-20	DSM-5	8	0.061
Luo (2022) [24]	China	28,689	adolescents	IGDS9-SF	DSM-5	8	0.046
Burén (2023) [26]	Sweden and Italy	995	adult	GSMQ-9	DSM-5	7	0.018
She (2022) [32]	China	1200	adolescents	DSM-5IGD Checklist	DSM-5	7	0.124
Alhamoud (2022) [29]	Saudi	726	adolescent	IGDS9-SF	DSM-5	9	0.219
Cao (2022) [42]	China	1805	adult	DSM-5 IGD Checklist	DSM-5	7	0.075
Malak (2023) [43]	Jordan	1020	adult	IGD-20	DSM-5	9	0.122
Alsunni (2022) [27]	Saudi Arabia	843	adult	DSM-5 IGD Checklist	DSM-5	9	0.215
Huang (2022) [25]	China	10,479	adolescent	IGDS9-SF	DSM-5	10	0.032
Siste (2022) [41]	Indonesia	1233	adult	IGDT-10	DSM-5	8	0.019
Alghamdi (2023) [34]	Saudi Arabia	391	adolescent	IGD-20	DSM-5	9	0.035
Shouman (2023) [28]	Egypt	870	adult	IGDSF-9	DSM-5	9	0.060
Khrad (2022) [35]	Saudi Arabia	306	adult	IGD-20	DSM-5	8	0.101
Deng (2024) [37]	China	9306	adolescent	IGD-20	DSM-5	9	0.018
Rafiemane (2023) [31]	Iranian	791	adult	IGDT-10	DSM-5	9	0.037
Mohamed (2023) [34]	Malaysia	5290	adolescents	IGDS9-SF	DSM-5	10	0.035
Chen (2023) [39]	China	3381	adult	GADIS-A	ICD-11	8	0.019
Mazaherizade (2022) [40]	Iran	260	adult	GADIS-A	ICD-11	8	0.042
Nabi Nazari (2023) [38]	Russian	933	adolescent	GADIS-A	ICD-11	8	0.040
Wu (2023) [45]	China	608	adult	GADIS-A	ICD-11	9	0.039
Grosemans (2023) [30]	Belgium	2289	adolescent	GADIS-A	ICD-11	7	0.025

**Table 2:** Subgroup analyses.

Variable	Subgroup	k	Prevalence	95% CI		*p* Significance Test(s)
Study location	Asia	19	0.075	0.063	0.086	*p* < 0.01 **
	European	3	0.026	0.016	0.037	
Sample size	>1000	11	0.049	0.038	0.06	*p* < 0.01 **
	<1000	11	0.097	0.062	0.132	
Adolescents/Adult						
	Adolescents	10	0.071	0.056	0.085	*p* = 0.651
	Adult	12	0.065	0.045	0.085	
Diagnostic criteria	DSM-5	17	0.079	0.066	0.092	*p* < 0.01 **
	ICD-11	5	0.030	0.021	0.039	
QS	<9	12	0.061	0.047	0.075	*p* = 0.115
	≥9	10	0.079	0.061	0.097	
Scale	DSM-5 IGD Checklist	3	0.137	0.066	0.208	*p* < 0.01 **
	IGD-20	6	0.102	0.054	0.149	
	IGDS9-SF	4	0.066	0.048	0.083	
	GSMQ-9	1	0.018	0.010	0.026	
	IGDT-10	2	0.027	0.009	0.044	
	GADIS-A	5	0.030	0.021	0.039	
	IGDSF-9	1	0.060	0.044	0.076	

Abbreviations: DSM-5 IGD checklist: Diagnostic and Statistical Manual of Mental Disorders, Fifth Edition, Internet Gaming Disorder Checklist; IGD-20: Internet Gaming Disorder-20 Test; IGDS9-SF: Internet Gaming Disorder Scale-Short Form; GSMQ-9: Game Addiction Scale for Adolescents-9; IGDT-10: Internet Gaming Disorder Test-10; GADIS-A: Gaming Addiction Identification Scale for Adolescents; IGDSF-9: Internet Gaming Disorder Scale for Adolescents—Short Form. ** *p* < 0.01.

## Data Availability

Data are available from the corresponding author upon reasonable request.
